# Urinary soluble CD163 is useful as “liquid biopsy” marker in lupus nephritis at both diagnosis and follow-up to predict impending flares

**DOI:** 10.1016/j.jtauto.2024.100244

**Published:** 2024-06-20

**Authors:** Yves Renaudineau, Dominique Chauveau, Stanislas Faguer, Antoine Huart, David Ribes, Gregory Pugnet, Laurent Sailler, Thibaut Jamme, Emmanuel Treiner, Françoise Fortenfant, Chloé Bost, Caroline Carlé, Julie Belliere

**Affiliations:** a Immunology Department Laboratory, Referral Medical Biology Laboratory, Institut Fédératif de Biologie, Toulouse University Hospital Center, France; b INFINITy, Toulouse Institute for Infectious and Inflammatory Diseases, INSERM U1291, CNRS U5051, University Toulouse III, Toulouse, France; c Department of Nephrology and Organ Transplantation, Referral Centre for Rare Kidney Diseases, University Hospital of Toulouse, INSERM U1297, Toulouse, France; d Internal Medicine, University Toulouse III, Toulouse, France, Biochemistry, Toulouse University Hospital, Toulouse, France; e Biochemistry Department Laboratory, Institut Fédératif de Biologie, Toulouse University Hospital Center, France

**Keywords:** lupus nephritis, Biomarkers, Urinary soluble CD163, Flare, Remission, End stage kidney disease

## Abstract

Lupus nephritis (LN) diagnosis and follow-up requires noninvasive biomarkers. Therefore, the added value of coupling the urinary soluble (s)CD163/creatinuria ratio with serological markers was evaluated in a real-world clinical practice. To this end, a monocentric and retrospective study was conducted in 139 SLE patients with biopsy-proven nephritis having an active LN (LN-A, n = 63 with a positive SLEDAI-renal score) or inactive (n = 76), as well as 98 non-renal SLE patients. The urinary sCD163/creatinuria ratio outperformed serological markers for predicting LN-A (AUC>0.972; p < 10^−4^ with a 100 % specificity threshold fixed at 320 ng/mmol), and for monitoring renal activity allowing prediction of impending flares and remissions in follow-up (AUC = 0.789, p < 10^−4^). LN-A patients with an elevated spot proteinuria/creatinuria ratio (p = 8 × 10^−6^) and sCD163/creatinuria ratio (p = 10^−3^) were at risk for developing end-stage kidney disease but sCD163/creatinuria ratio cannot substitute kidney biopsy to discriminate LN-A from other glomerulonephritis. Among serological markers (n = 14), anti-dsDNA and anti-C1q antibodies (Abs) (AUC>0.750 versus non-LN patients, and AUC>0.640 versus LN-IR patients) best predicted LN-A, and higher levels were retrieved in class III/IV proliferative LN-A. In multivariate logistic regression analysis, the urinary sCD163/creatinuria ratio remained the only statistically significant biomarker to predict LN-A (p < 0.001). In conclusion, and as compared to classical serological markers, the urinary sCD163/creatinuria ratio provides an additional parameter for monitoring LN patients.

## Highlights

• Classical biomarker performances are limited in lupus nephritis (LN).
• There is added value in using urinary sCD163/creatinuria ratio in LN.
• Urinary sCD163/creatinuria ratio variations predict impending flares and remissions.
• An elevated sCD163/creatinuria level is a risk factor for ESKD.

## 1 Introduction

Lupus nephritis (LN), which affects 30–60 % of systemic lupus erythematosus (SLE) patients, is associated with increased morbidity, mortality, and can lead to relapses (35 %) and end stage kidney disease (ESKD, 5–20 %) [1]. As a consequence, when treating active LN (-A), several objectives have to be considered in order to: (i) normalize the kidney functions; (ii) limit relapses; (iii) prevent ESKD; and (iv) reduce systemic side-effects [2]. Therefore, to propose the most suitable treatment, an accurate diagnosis is necessary and for that the gold standard remains the percutaneous kidney biopsy, which helps to define the histological lesion type, the presence of any active/chronic glomerular injury, as well as vascular and/or tubulointerstitial lesions [3]. This applies in particular for proliferative LN (class III/IV ± V), which presents a more aggressive course as compared to membranous LN (class V) and mesangial LN (class I/II), and for the former, treatment needs to be more aggressive although long-term remission is infrequently achieved [4,5]. However, individuals from the same LN class may respond differently, class-switching may occur during therapies, and this implies consideration of a second biopsy and obtaining a panel of laboratory findings [6]. Percutaneous kidney biopsy is in addition associated with bleeding complications such as macroscopic hematuria (3.5 %), post-biopsy hematoma (11.6 %), erythrocyte transfusion (0.9 %), and rarely nephrectomy (0.01 %) or death (0.02 %) and it has been further reported that acute kidney injury increases the risk of bleeding [7,8]. Moreover, kidney biopsy is contraindicated in a number of patients including presence of a single kidney (especially after renal cancer), blood coagulation disorders, and antiplatelet and/or anticoagulant therapies. To circumvent kidney biopsy limitations and contraindications, a large panel of biomarkers is currently used to monitor LN patients including SLE-associated autoantibodies (Abs), complement parameters, and urinary biomarkers. Since these classical biomarkers can present unsatisfactory results, additional “liquid biopsy” biomarkers are mandatory, but their expected performance results have to be established in real world practices.

In the last two decades, and although not completely understood, LN physiopathology has been revealed to be more complex than initially proposed with kidney injury resulting directly from nucleic acid-immune complex deposition and subsequent complement activation. In addition to formation of immune complexes, it has been reported, on one hand, that part of the spectrum of anti-dsDNA/chromatin Abs cross-react with glomerular cells that can be activated [[9], [10], [11]] and, on the other hand, that the half-life of the nucleic acid-immune complexes present in the kidney is abnormally increased. At least three processes contributing to reduce renal nucleic acid-immune complexes are altered in LN, a defective renal DNAse activity that can be affected due to innate or acquired deficiency of DNASE1L3 [12,13], a reduction in the opsonization process [14], and the shift from phagocytic macrophages into non-phagocytic macrophages M2 type bearing the cell surface cluster of differentiation (CD)163 that can be cleaved and released in the urine in response to an inflammatory stimulation [15]. Glomerular macrophages M2 cell number and urinary sCD163 are correlated with proteinuria, disease activity, and cellular crescents that may evolve to fibrosis and in turn ESKD [16,17]. In addition, macrophages M2 from LN kidneys can promote the formation of extracellular autoreactive B cell Ab producing aggregates. Last but not least and when present, anti-C1q Abs are able to form super-immune complexes by recruiting granulocytes that in turn amplify renal injury [18].

Accordingly, and in order to test the added value to explore the urinary sCD163 factor as “liquid kidney biopsy” biomarker in a large panel of serological and urinary LN/SLE biomarkers, 237 SLE patients were retrospectively selected. For these biomarkers, their capacity to discriminate against active LN (LN-A) was tested as well as their performance to explore active/chronic disease variations during follow-up.

## 2 Material and methods

### 2.1 Patient selection

In this cross-sectional and follow-up study, 237 SLE patients investigated for SLE-associated biomarkers in a tertiary laboratory of immunology at the request of clinicians from the Department of Nephrology and Organ Transplantation and Internal Medicine Department (CHU Toulouse, France) were initially selected from January 2022 (at anti-C1q Ab introduction in the panel) to March 2024 (urinary sCD163 was introduced in January 2023). Among them, 93 LN patients, including 36 with active LN (-A) and 57 with inactive/remission LN (-IR), were tested at two time points during follow-up (median delay: 209 days with an interquartile [IQ] of 141–378 days). All SLE patients met the 2019 ACR/EULAR classification criteria [19], and biopsy-proven LN patients were classified according to the 2018 revision of the ISN/RPS criteria [3]. Information collected from medical records included age, sex, disease duration, renal activity score (SLEDAI-R) that included from the SLEDAI-2K score the four kidney-related parameters: proteinuria, hematuria, pyuria and urinary casts [20], the estimated glomerular filtration rate (eGFR) [21], clinical presentation, and current treatments. The study was conducted according to the guidelines of the declaration of Helsinki, participants were informed and gave their consent, and the related cohorts were approved by the ethics committee in France (CPP) under the references RC31/21/0154, Molecular Prediction of Development, Progression or Complication of Kidney, Immune or Transplantation-related Diseases (Nephrogen) and 2021-A03236-35 (ESSAi).

### 2.2 Immunological parameters

The immunological panel for SLE-associated biomarkers included: (i) the complement fractions C3, C4 (Cobas 500**®**, Roche Diagnostics GmBH, Germany) and 50 % concentration haemolytic (CH50; SpaPlus**®**, The Binding Site, Birmingham, UK); (ii) IgG anti-double stranded (ds)DNA and anti-chromatin Abs (Bioplex**®**, Biorad, Hercules, CA); (iii) IgG anti-extractable nuclear (ENA) Abs against sicca syndrome (SS)A 52 kDa, SSA 60 kDa, SSB, Smith (Sm), Sm-ribonucleoprotein (RNP), RNP A, and RNP 68 kDa antigens (Bioplex**®**); (iv) IgG anti-ribosomal Abs (Bioplex**®**); (v) IgG anti-C1q Abs (Quanta-lite**®**, Werfen, Barcellona, Spain); and (vi) the spot urinary sCD163 (ELLA**®**, Bio-techne, Minneapolis, MN) that was evaluated together with proteinuria and creatinuria (Cobas 500**®**) in order to express sCD163 as a ratio to creatinuria, proteinuria, or not as well as the ratio of urine protein excretion to creatinine or PCR [[22], [23], [24], [25], [26]]. Cut-offs were fixed as recommended by the providers (C3 low <0.72 g/L; C4 low <0.11 g/L, CH50 low <31 %; anti-dsDNA Abs ≥10 international units [IU]/mL, anti-ENA/-Ribosomal/-Chromatin Abs ≥1.0 arbitrary units [AU]mL, and anti-C1q Abs ≥20 AU/mL) with the exception of the normalized urinary sCD163/creatinuria ratio as no consensus existed.

### 2.3 Statistical analysis

Quantitative data are presented as median and IQ 25th-75th percentile, and analyzed using non-parametric tests with Dunn's test applied for post-hoc multiple comparisons in analysis of variance (ANOVA) when necessary. Categorical data were analyzed using Fisher's exact test. Receiver operating characteristic (ROC) curves were generated to determine the area under the curve (AUC) that was interpreted as follow: very good (0.9<AUC<1), good (0.7<AUC≤0.89), weak (0.6<AUC ≤0.69), and useless (≤0.59). When necessary, sensitivity, specificity, positive predictive value (PPV), negative predictive value (NPV), and related 95 % confidence intervals (CI) were calculated. The Spearman's rank-correlation test was used to compare techniques during follow-up, and the absolute rho (r) interpreted as follows: very good (±0.8<rho ≤±1), good (±0.5<rho ≤±0.79), weak (±0.2<rho ≤±0.49), and useless (rho ≤±0.39). The multivariate logistic regression was performed to assess the relation between LN-A and the explanatory variables (cCD163/creatinuria ratio, anti-dsDNA Abs, anti-C1q Abs, and C3 levels). The alpha risk was set to 0.05 and statistical analysis performed using GraphPad Prism 10.2 (La Jolla, CA) and EasyMedStat (version 3.34.1; www.easymedstat.com).

## 3 Results

### 3.1 SLE demographic data

For this study 237 SLE patients were recruited, including 139 SLE patients with biopsy-proven nephritis and dichotomized into LN-A when the SLEDAI-R score was positive (n = 63) or otherwise into LN-IR (n = 76), as well as 98 non-renal SLE patients (non-LN) (Table 1). A female predominance was reported (204 females vs 33 males), the median age was 41 years old (IQ: 31–53 years), and time from SLE diagnosis was similar between groups (12 years, IQ: 5–21 years).

**Table 1:** Patients’ characteristics.

	LN-A	LN-IR	Non-LN	Statistics LN-A vs LN-IR	Statistics LN-A vs non-LN
Cross-sectional (follow-up), number	63 (36)	76 (57)	98 (0)	–	–
Age (years), median [IQ]	38 [30–47]	39 [30–54]	45 [35–56]	0.999	0.020
Sex, F:M	52:11	71:5	81:17	0.061	0.999
Time from SLE diagnosis (years)	12 [[3], [4], [5], [6], [7], [8], [9], [10], [11], [12], [13], [14], [15], [16], [17], [18], [19]]	13 [[7], [8], [9], [10], [11], [12], [13], [14], [15], [16], [17], [18], [19], [20], [21], [22]]	13 [[6], [7], [8], [9], [10], [11], [12], [13], [14], [15], [16], [17], [18], [19], [20], [21], [22]]	0.212	0.155
Time from last PRB (years)	0 [0–3]	5 [[2], [3], [4], [5], [6], [7], [8], [9]]	–	<10^−4^	–
SLEDAI-2K score	9 [[6], [7], [8], [9], [10], [11], [12], [13], [14], [15], [16]]	2 [0–2]	1 [0–3.5]	<10^−4^	<10^−4^
SLEDAI-R score	8 [[4], [5], [6], [7], [8], [9], [10], [11], [12]]	0	0	<10^−4^	<10^−4^
Renal biopsy: I-II/III-IV/V	3/52/8	7/61/8	–	0.617	–
Mucocutaneous	48/63 (76.2 %)	65/76 (85.5 %)	85/98 (86.7 %)	0.192	0.093
Musculoskeletal	49/63 (77.8 %)	59/76 (77.6 %)	77/98 (78.6 %)	0.999	0.125
Hematological	22/63 (34.9 %)	26/76 (34.2 %)	21/98 (21.4 %)	0.999	0.069
Cardiorespiratory	10/63 (15.9 %)	18/76 (23.7 %)	23/98 (23.5 %)	0.293	0.318
Neurological	12/63 (19.0 %)	11/76 (14.5 %)	15/98 (15.3 %)	0.499	0.666
APS	7/63 (11.1 %)	15/76 (19.7 %)	22/98 (22.4 %)	0.245	0.092
HCQ	49/63 (77.8 %)	63/76 (82.9 %)	66/98 (67.3 %)	0.520	0.211
GC	41/63 (65.1 %)	28/76 (36.8 %)	30/98 (30.6 %)	0.001	<10^−4^
IS/AM	42/63 (66.7 %)	44/76 (57.9 %)	28/98 (28.6 %)	0.300	<10^−4^
Biologics	23/63 (36.5 %)	24/76 (31.6 %)	16/98 (16.3 %)	0.591	0.005
C3 low (<0.72 g/L)	23/62 (31.1 %)	8/76 (10.5 %)	7/93	0.0004	<10^−4^
C4 low (<0.11 g/L)	24/62 (38.7 %)	14/76 (18.4 %)	20/93	0.01	0.03
CH50 low (<31 %)	19/62 (30.6 %)	7/76 (9.2 %)	14/93	0.002	0.03
Anti-dsDNA Abs (≥10 IU/mL)	45/62 (72.6 %)	36/76 (47.4 %)	34/96	0.003	<10^−4^
Anti-Chromatin Abs (≥1 AU/mL)	47/62 (75.8 %)	30/76 (39.5 %)	36/95	<10^−4^	<10^−4^
Anti-C1q Abs (≥20 AU/mL)	34/57 (59.6 %)	20/65 (30.8 %)	17/80	0.002	<10^−4^
sCD163/creatinuria ratio (≥320 ng/mmol)	50/50 (100 %)	5/67 (7.5 %)	9/80	<10^−4^	<10^−4^

**Abbreviations:** LN: lupus nephritis; A: active; IR: inactive/remission; SLE: systemic lupus erythematosus; IQ: interquartile; F: female; M: male; PRB: percutaneous renal biopsy; SLEDAI-2K: SLE disease activity score 2000; SLEDAI-R: SLEDAI renal score; APS: antiphospholipid syndrome; HCQ: hydroxychloroquine; GC: glucocorticoids; IS/MS: immunosuppressant/antimetabolite drug; CH50: 50 % concentration haemolytic; Abs: IgG autoantibodies.

LN-A and LN-IR groups were similar when considering sex-ratio, renal biopsy class distribution, non-renal clinical manifestations, and therapies except for glucocorticoid intake (p = 0.001). The non-LN group was characterized, as compared to the LN-A group, by a higher age at inclusion (p = 0.02), a lower disease activity (SLEDAI-2K, p < 10^−4^), and less aggressive therapies with regard to glucocorticoids, immunosuppressant/antimetabolite, and add-on biotherapies usage (p < 0.005 for all).

### 3.2 Serological markers and urinary sCD163/creatinuria ratio utility in active LN

**Fig. 1:**
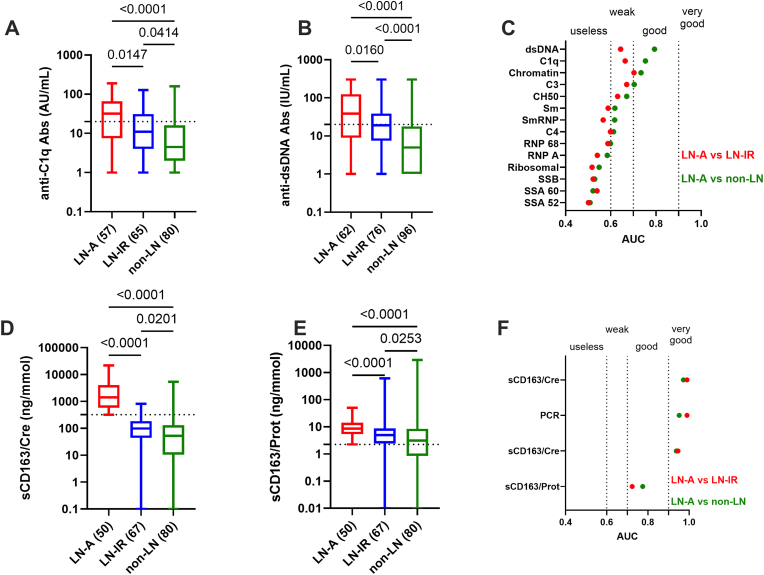
**Serological markers and urinary soluble (s)CD163/creatinuria utility for active lupus nephritis (LN-A). A/B:** Anti-C1q and anti-dsDNA autoantibody (Ab) levels in patients with LN-A *versus* inactive/remission LN (LN-IR) and non-renal patients (non-LN), the number of patients is added in brackets. The dotted lines reflect the manufacturers' cut-offs for anti-C1q Abs (20 arbitrary units [AU]/mL) and anti-dsDNA Abs (10 international units [IU]/mL). **C:** Area under the curve (AUC) values obtained from receiver operating characteristic (ROC) curves of 11 Abs (targeting C1q, dsDNA, chromatin, SSA 52 kDa, SSA 60 kDa, SSB, Sm, SmRNP, RNP 68, RNP A, and ribosomal antigens) and 3 complement parameters (C3, C4 and CH50) comparing LN-A patients from LN-IR patients (red dots) or from non-renal SLE patients (green dots). **D/E:** Urinary sCD163/creatinuria (sCD163/Cre) and sCD163/proteinuria (sCD163/Prot) ratio levels in patients with LN-A versus LN-IR and non-LN. The dotted line reflects the 100 % specificity threshold fixed at 320 ng/mmol for urinary sCD163/Cre ratio and at 2.2 ng/g for urinary sCD163/Prot ratio. **F:** AUC values obtained from ROC curves of urinary biomarkers including spot protein excretion to creatinine ratio (PCR) and sCD163 expressed as a ratio to creatinuria (sCD163/Cre), proteinuria (sCD163/Prot), or not (sCD163 total). LN-A patients are compared to the LN-IR patients (red dots) or to the non-renal SLE patients (green dots). **F:** ROC curves of the 4 urinary biomarkers. The *p* values are indicated.

As presented in Fig. 1A/B, both anti-C1q Ab and anti-dsDNA Ab levels were effective to discriminate LN-A from LN-IR (p = 0.02, both) and from non-LN patients (p < 10^−4^, both). Next, and to test anti-C1q/dsDNA Ab performances according to the classical serological panel used to follow SLE patients, a ROC approach was selected to rank the different biomarkers (Fig. 1C). Among them, 4 parameters were effective to discriminate with good performances LN-A non-LN patients (0.7<AUC<0.89): anti-dsDNA Abs (AUC = 0.793; p < 10^−4^), followed by anti-C1q Abs (AUC = 0.753; p < 10^−4^), anti-Chromatin Abs (AUC = 0.733; p < 10^−4^), and the complement C3 fraction (AUC = 0.704; p < 10^−4^). For these 4 parameters, capacity to discriminate LN-A from LN-IR remained at weaker performances (0.64<AUC<0.71). Of note, hypocomplementemic urticarial vasculitis (HUV) or McDuffie syndrome with anti-C1q Ab positivity was present in 3 cases [range: 41–191 UA/mL] including 1 LN-A and 2 non-LN patients.

Regarding urinary sCD163 tested in SLE patients (Fig. 1D), the ratio with creatinuria (sCD163/creatinuria) was highly effective to discriminate LN-A (n = 50) both from LN-IR (n = 67; AUC = 0.990 and p < 10^−4^) and from non-LN (n = 80; AUC = 0.972 and p < 10^−4^). The ROC curves between LN-A with LN-IR and between LN-A with non-LN patients were further used to demonstrate that sCD163/creatinuria ratio performances were higher than those obtained with the spot urine PCR (AUC = 0.989 and 0.951, respectively), the unnormalized value of sCD163 (sCD163 total, AUC = 0.946 and 0.938, respectively), and even more with the sCD163 normalized to proteinuria (AUC = 0.723 and 0.775, respectively) (Fig. 1E/F). To our knowledge no study has addressed the clinical threshold for urine sCD163/creatinuria ratio to discriminate LN-A in real world cases, therefore 320 ng/mmol was selected from the ROC curve as this value presented a specificity of 100 % (95 % CI: 92.9–100 % for both), a sensitivity of 92.5 % (95 % CI: 83.7–96.8 %) and 85.0 % (95 % CI: 75.6–91.2 %), a PPV of 90.9 % (95 % CI: 80.4–96.1 %) and 84.7 % (95 % CI: 73.5–91.8 %), and a NPV of 100 % for both (95 % CI: 94.2–100 % and 94.9–100 %) to differentiate LN-A from LN-IR and from non-LN groups, respectively. At 100 % specificity, the threshold was 37.5 ng for total sCD163 (sensitivity of 4 % versus LN-IR and 19.5 % versus non-LN), and 2.2 ng/g for the sCD163/proteinuria ratio (sensitivity of 22 % versus LN-IR and 39 % versus non-LN).

Altogether and to differentiate LN-A, urinary sCD163/creatinuria ratio outperforms serological and urinary biomarkers, while this biomarker was ineffective to differentiate LN-IR patients from non-renal SLE patients. In multivariate logistic regression analysis, the urinary sCD163/creatinuria ratio remained the only statistically significant biomarker to predict LN-A (p < 0.001), which was not the case for serological biomarkers (anti-dsDNA Abs, anti-C1q Abs and complement C3) (data not shown).

### 3.3 Effect of proteinuria on urinary sCD163/creatinuria ratio determination

**Fig. 2:**
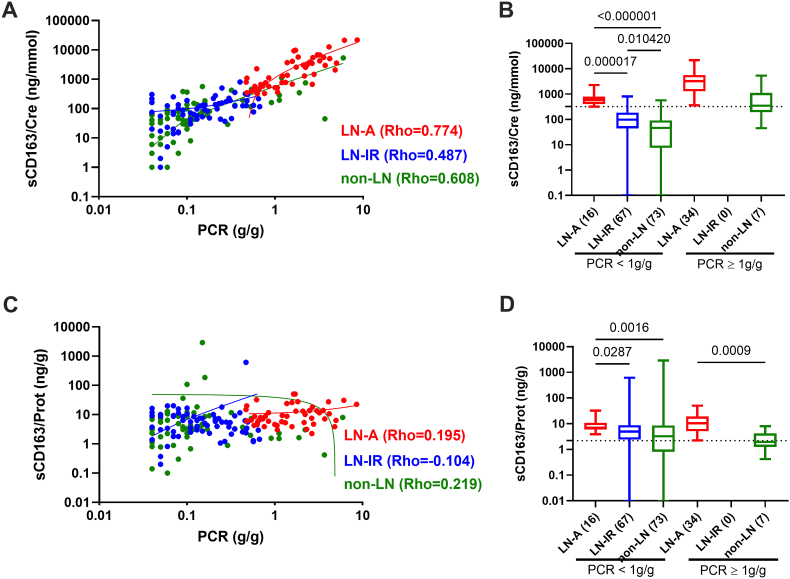
**Proteinuria effect on urinary sCD163 estimation. A:** Correlation between spot urinary sCD163/creatinuria (sCD163/Cre) with protein excretion to creatinine ratio (PCR) in patients with active lupus nephritis (LN-A in red, n = 50), inactive/remission LN (LN-IR in blue, n = 60), and non-LN patients (non-LN in green, n = 80). Spearman's rho values are indicated. **B:** Urinary sCD163/Cre levels in patients with LN-A versus LN-IR and non-LN according or not to the presence of a proteinuria (PCR≥ 1 g/g), p values are indicated when significant. **C:** Correlation between spot urinary sCD163/proteinuria (sCD163/Prot) with PCR. **D:** Urinary sCD163/Prot levels in LN-A, LN-IR and non-LN patients according or not to the presence of a proteinuria (PCR≥ 1 g/g).

As reported in patients with active anti-neutrophil cytoplasmic antibody-associated vasculitis [27,28], a non-specific leakage of sCD163 from blood may occur as the proteinuria increases and this can be controlled by using the urinary sCD163/proteinuria ratio. Indeed (Fig. 2), a strong correlation was observed between spot PCR and urinary sCD163/creatinuria ratio in LN-A patients (PCR median: 1417 ng/mmol [IQ: 581–4120]; Spearman's rho = 0.774, p < 10^−4^), in LN-IR patients (PCR median: 98 ng/mmol [IQ: 44–186]; rho = 0.487, p = 0.05), and in non-LN patients (PCR median: 52 ng/mmol [IQ: 10.5–131]; rho = 0.608, p < 10^−4^). Such correlation with PCR, was not retrieved when using the urinary sCD163/proteinuria ratio (−0.104<rho<0.219). With the limitation that the number of non-LN patients with a PCR≥1 g/g (n = 7) was reduced and related to biopsy proven non-LN glomerulonephritis (IgA deposition x2, post-infectious, minimal change nephropathy, and focal segmental glomerulosclerosis) or a chronic kidney disease at end stage (CDK5, n = 5), for these limited cases we confirmed at PCR≥1 g/g a better discrimination with LN-A when using the urinary sCD163/proteinuria ratio instead of the sCD163/creatinuria ratio (p = 0.0009 versus p = 0.09), which needs further investigation.

### 3.4 Histological lesions in LN-A and related biomarkers

As a more aggressive therapy is recommended in cases of proliferative class III/IV LN-A, it's then important to evaluate serological and urinary biomarkers capacity of substituting for kidney biopsy to discriminate proliferative (class III/IV ± V, n = 47 biomarkers tested except n = 39 for urinary sCD163) from isolated membranous LN-A (class V, n = 8 except for urinary sCD163: n = 6). For statistical purposes and due to incomplete data, the three LN-A patients with mesangial class II glomerulonephritis were not included in the analysis (urinary sCD163/creatinuria ratio tested for one: 361 ng/mmol).

**Fig. 3:**
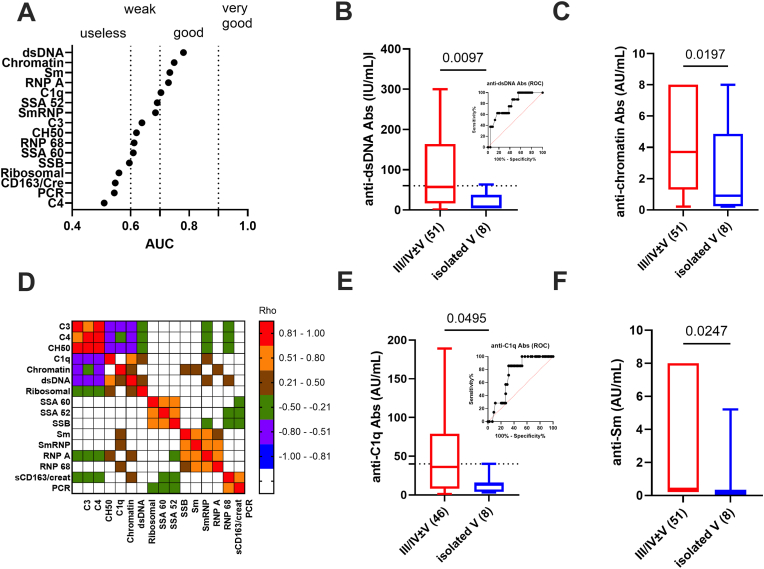
**Anti-C1q/dsDNA antibodies (Ab) levels but not urinary sCD163/creatinuria levels discriminate proliferative from membranous active lupus nephritis (LN-A). A:** Area under the curve (AUC) values obtained from receiver operating characteristic (ROC) curve of 11 IgG autoantibodies (targeting C1q, dsDNA, chromatin, SSA 52 kDa, SSA 60 kDa, SSB, Sm, SmRNP, RNP 68, RNP A, and ribosomal antigens), 3 complement parameters (C3, C4 and CH50), and 2 urinary parameters (spot protein to creatinuria ratio [PCR], and urinary sCD163 normalized to creatinuria) comparing proliferative LN-A (class III/IV ± V) from membranous LN-A (isolated class V). Biomarkers presenting good (0.7 ≤ AUC<0.89, p < 0.05) performance to discriminate proliferative LN-A are presented: anti-dsDNA Abs (**B**), anti-chromatin Abs (**C**), anti-C1q Abs (**D**), and anti-Sm Abs (**E**). The dotted line reflects the histological cut-offs for anti-dsDNA Abs (60 international units [IU]/mL, 100 % sensitivity) and anti-C1q Abs (40 arbitrary units [AU]/mL, 100 % sensitivity), the number of patients is added in brackets, ROC curves are included for anti-dsDNA and anti-C1q Abs, and *p* values are indicated when significant. **F:** Spearman's correlation matrix between the 16 biomarkers to test their associations in LN-A.

To this end, ROC curves were used to rank the biomarker capacity to discriminate proliferative from membranous LN-A after excluding any potential effect of treatment (Fig. 3A and data not shown). Four serological biomarkers exhibited good performance in discriminating proliferative class III/IV ± V from isolated membranous class V (AUC≥0.7 and p ≤ 0.05): anti-dsDNA Abs (AUC = 0.819, p = 0.0035), anti-chromatin Abs (AUC = 0.735, p = 0.03), anti-C1q Abs (AUC = 0.730, p = 0.04), and anti-Sm Abs (AUC = 0.727, p = 0.045) (Fig. 3B/C-E/F). Using a sensitivity of 100 % for setting the histological threshold, a cut-off of 60 IU/mL was considered for anti-dsDNA Abs (specificity: 44.4 %, 95 % CI: 31.4–58.8 %; sensitivity: 100 %; 95 % CI: 67.6–100 %) and of 40 AU/mL for anti-C1q Abs (specificity: 47.7 %, 95 % CI: 33.8–62.1 %; sensitivity: 100 %; 95 % CI: 64.6–100 %). These thresholds can help but can't substitute for a kidney biopsy to discriminate proliferative from membranous LN. Regarding urinary parameters (PCR, sCD163/creatinuria ratio, and sCD163/proteinuria ratio), none of them were significantly effective in discriminating proliferative from membranous LN-A. Next and to test interdependence between biomarkers (Fig. 3D), a Spearman's correlation matrix was done revealing five partially independent clusters: the complement cluster (C3, C4, CH50: rho³0.8), the anti-dsDNA/Chromatin/C1q/ribosomal Ab cluster (0.2<rho<0.76), the anti-SSA/SSB Ab cluster (0.52<rho<0.65), the anti-Sm/RNP Ab cluster (0.46<rho<0.78), and the urinary cluster (rho = 0.79). This further reinforces the independence between serological and urinary biomarkers.

### 3.5 Serological and urinary sCD163/creatinuria ratio levels according to SLEDAI-R

**Fig. 4:**
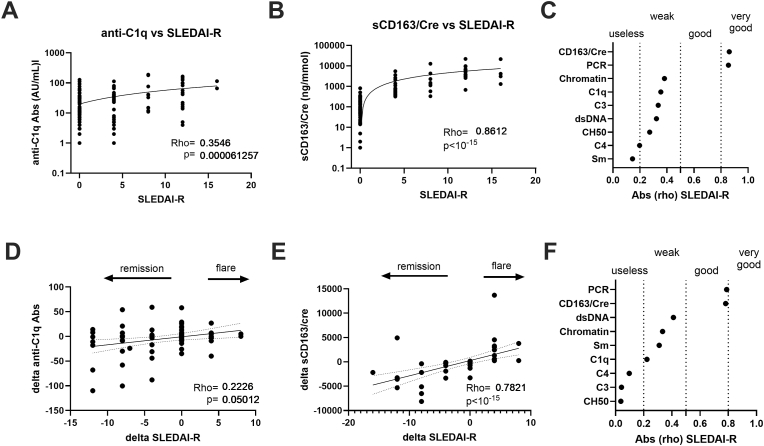
**Serological markers and even more urinary sCD163/creatinuria (sCD163/Cre) levels are correlated with renal disease activity (SLEDAI-R) in lupus nephritis (LN) patients. A:** Weak correlation between anti-C1q Abs and SLEDAI-R in a cross-sectional analysis (n = 122), r and p values are reported. **B:** Very good correlation between urinary sCD163/Cre levels and SLEDAI-R in a cross-sectional analysis (n = 117). **C:** Spearman's absolute rho [abs (rho)] values obtained between SLEDAI-R and urinary biomarkers (spot protein to creatinuria ratio [PCR], and urinary sCD163/Cre) or serological biomarkers (IgG anti-dsDNA Abs, anti-chromatin Abs, anti-C1q Abs, and complement C3, C4, CH50). **D:** Changes (delta) in anti-C1q Abs correlated weakly in LN patients (n = 92) with the evolution of disease activity at follow-up (delta SLEDAI-R). **E:** in contrast, delta-urinary sCD163/Cre levels presented a good correlation with delta SLEDAI-R (n = 61). **F:** Spearman's absolute rho (abs (rho)) values obtained between delta SLEDAI-R and changes (delta) in urinary and serological biomarkers during LN follow-up. According to the variations in SLEDAI-R, delta SLEDAI-R is considered to reflect therapeutic response/remission (negative delta value), an inactive/stable disease (unchanged delta value), and flare (positive delta value).

With regard to the renal disease activity assessed from the cross-sectional analysis in 139 LN patients (63 with LN-A and 76 with LN-IR), a weak correlation was reported between SLEDAI-R and anti-C1q Abs (rho = 0.354; p = 6 × 10^−5^), while a very good correlation characterized urinary sCD163/creatinuria ratio levels (rho = 0.861; p < 10^−15^) (Fig. 4A–B). As compared to the other serological biomarkers (Fig. 4C), anti-C1q Ab performances were close to those obtained with anti-Chromatin Abs (rho = 0.381) and higher than those reported with complement parameters (−0.335<rho < −0.197) and anti-Sm Abs (rho = 0.145). When comparing urinary biomarkers, close performances were reported between the sCD163/creatinuria ratio and PCR (rho = 0.861 *versus* 0.854, respectively).

Next, variations in levels of serological markers and the urinary sCD163/creatinuria ratio were assessed at two time points in 93 LN patients (36 with LN-A and 57 with LN-IR) and variations in their SLEDAI-R used to consider therapeutic response/remission (negative delta SLEDAI-R), inactive/stable disease (unchanged delta), and SLE flare (positive delta) (Fig. 4D/F). Using this approach, changes in anti-C1q Abs were weakly associated with SLEDAI-R variations (rho = 0.222; p = 0.05) as compared to a good capacity of the urinary sCD163/creatinuria ratio to predict SLEDAI-R variations (rho = 0.782; p < 10^−15^). Results obtained in the longitudinal analysis confirmed those obtained in the cross-sectional analysis and supported the utility of the urinary sCD163/creatinuria ratio as a surrogate for SLEDAI-R disease activity.

### 3.6 Serological and urinary sCD163/creatinuria ratio levels according to eGFR

**Fig. 5:**
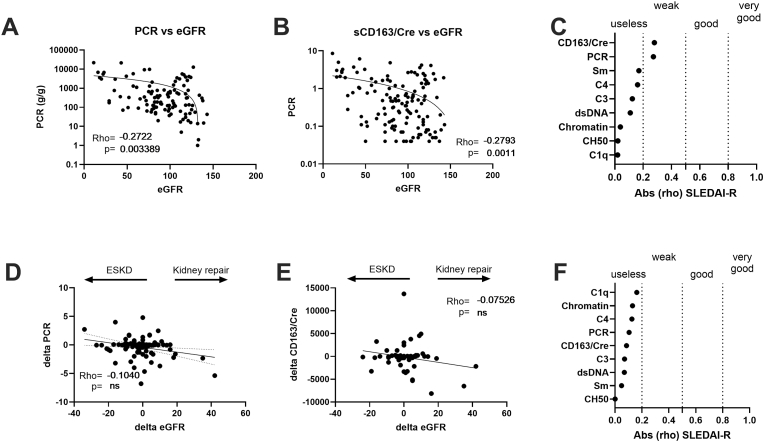
**Urinary sCD163/creatinuria (sCD163/Cre) levels but not serological markers are weakly correlated with the evolution of the renal function (eGFR) in lupus nephritis (LN) patients. A-B:** In cross-sectional analysis, a weak correlation was found between eGFR and spot protein to creatinuria ratio (PCR, n = 139) (**A**), and with urinary sCD163/Cre ratio levels (n = 117) (**B**). **C:** Spearman's absolute rho [abs (rho)] values obtained between eGFR and urinary biomarkers or serological biomarkers. **D-F:** No significant correlation (p < 0.05) between urinary and serological biomarkers with delta eGFR during LN follow-up. According to the variations in eGFR, delta eGFR is considered to reflect end stage kidney disease evolution (ESKD, negative delta value), a stable evolution (unchanged delta value), and kidney repair with time (positive delta value).

The eGFR level is a sensitive indicator of renal function and a negative evolution predicts ESKD [29]. Accordingly, serological and urinary sCD163/creatinuria ratio levels were compared to eGFR levels in a cross-sectional analysis from 139 LN patients, with a weak association reported for PCR (rho = −0.291; p = 8 × 10^−6^), urinary sCD163/creatinuria ratio (rho = −0.228; p = 0.0015), and anti-Sm Abs (rho = 0.222; p = 0.001) (Fig. 5A–C). In the longitudinal analysis, variations in eGFR between the two time points were considered to reflect an ESKD evolution (negative delta eGFR), a stable kidney function (unchanged delta), and kidney repair (positive delta). With this approach, no association was retrieved with eGFR evolution and serological or urinary biomarkers. This supports the concept that an elevated level in urinary PCR and sCD163/Cre levels contributes to alter eGFR and in turn promotes ESKD, while variations during follow-up were not associated with eGFR improvement and renal repair at least in our study.

## 4 Discussion

In addition to the kidney biopsy, which is invasive and presents contraindications that restrict its usage, the detection of SLE-associated “liquid biopsy” biomarkers is important at the time of LN diagnosis, and part of them can be used to monitor LN activity and in turn to provide guidance for therapeutic response, flare, and ESKD evolution. The use of biomarkers is of further importance as there is a weak correlation between clinical presentation (isolated hematuria, or proteinuria, nephrotic syndrome) and histological presentation. Results from our study support the consideration of the urinary sCD163/creatinuria ratio as an independent factor in the list of classical LN biomarkers in addition to anti-dsDNA Abs, anti-C1q Abs, C3 complement fraction, PCR and eGFR.

A defective clearance of renal nucleic acid-immune complexes is central in LN pathophysiology, and part of this defect is related to the shift of phagocytic macrophages into non-phagocytic macrophages M2 (CD163+) having repair/injury activity [30]. Macrophages M2 represent the most abundant pro-inflammatory renal macrophage subtype that is located close to crescents, a sign of severe glomerular damage. To monitor renal macrophage M2 (CD163+) activity and as CD163 is shed from these cells, it was proposed to monitor the urinary 120 kDa transmembrane glycoprotein CD163 as a surrogate after normalization to creatinuria [16]. Indeed, normalization to creatinuria allows control of variations in the urine flow rate, explaining why urinary sCD163/creatinuria ratio determination performs better than total urinary sCD163 to discriminate LN-A as confirmed in our study. Another advantage to introducing urinary sCD163/creatinuria ratio determination as an LN biomarker is to overcome 24-h proteinuria and PCR limitations regarding tubular proteinuria (<25 kDa proteins) and the attribution of proteinuria to LN in a context of permanent renal damage, diabetes, hypertensive nephropathy, urinary tract infection, and other situations. Consequently, the urinary sCD163/creatinuria ratio used as a biomarker better predicts disease activity than PCR in our study, which is in agreement with previous studies [17,28,31]. We propose to fix the threshold for LN-A at 320 ng/mmol based on a specificity of 100 %, allowing the urinary sCD163/creatinuria ratio to discriminate LN-A in an SLE background. This is close to the <370 ng/mmol proposed to predict complete response in LN, and to the 250 ng/mmol value reported to discriminate active from inactive AAV associated with ANCA [27,28]. For the first time, we use a next-generation ELISA platform (e.g., ELLA), which is compatible with a routine use in clinical practice, compared to the previously published ELISA kits. This technology allows higher samples treatment rates and can be easily calibrated to standardize detection. However, urinary sCD163/creatinuria ratio determination suffers from limitations as highlighted in this study. First, glomerular basement membrane injury can lead to an overestimation of the urinary sCD163/creatinuria ratio level due to the leakage of circulating sCD163, and this phenomenon is starting to occur when PCR>1 g/g. As reported here, this can be retrieved associated with kidney failure (ESKD stage 5) or with biopsy proven non-LN glomeruloneprhitis in response to IgA deposition, infections, focal segmental glomerulosclerosis, and minimal change nephropathy. In patients with nephrotic syndrome and with severe loss of kidney function, urinary sCD163 is not a relevant biomarker. To circumvent this limitation [27], a normalization to proteinuria was proposed, but when applied to LN patients its value seems to be limited to elevated proteinuria levels (PCR>1 g/g) based on the fact that urinary sCD163/creatinuria ratio outperforms urinary sCD163/proteinuria ratio to discriminate LN-A. Second, we failed to confirm that urinary sCD163/creatinuria or sCD163/proteinuria ratios are significantly elevated in proliferative LN-A as previously reported [17]. This discrepancy may result in Zang's report to the non-consideration of the proteinuria as the mean PCR was 2 g/g in the class II/V group versus 3.5 g/g in the proliferative class III/IV group, which may affect the interpretation as reported in Fig. 2. Not explored in this study, others have reported that peripheral blood sCD163 performance was less than urinary sCD163 for disease activity in LN, but peripheral blood sCD163 can be used to predict non-renal lupus activity in SLE patients including cardiovascular disease, macrophage syndrome, and skin rash among others [[32], [33], [34]]. In addition to urinary sCD163/creatinuria ratio as reported in our study, the serum sCD163 level is also correlated with kidney end stage disease evolution [35].

A defect in the classical complement pathway is associated with SLE development and flares by impairment of the immune complex's clearance [36]. Indeed, the strongest genetic factor associated with SLE is noted to be C1q (93 % in homozygous) and an increased genetic risk is also reported with the other components of the classical pathway (C1r, C1s, C4, C2 and C3) [37]. Genetic defects are rare events and more often SLE patients develop a secondary C1q deficiency due to the presence of anti-C1q Abs with a prevalence ranging from 30 to 70 % and in this case an association with proliferative LN is reported [38], as well as an association with hypocomplementemic urticarial vasculitis (HUV) with 3 cases encountered in this study [39]. In patients from our cohort with LN, anti-C1q Ab and anti-dsDNA Ab detection performances were close in predicting LN-A, for suspecting a proliferative class III/IV ± V LN-A, and during follow-up to evaluate the therapeutic response and flares in agreement with previous studies [38,40]. Anti-C1q Abs are expressed in arbitrary units (AU) and the assigned positive cut-off fixed by Werfen's laboratory using healthy controls as reference (20 AU/mL) ought to have good capacity to discriminate LN-A from LN-IR and non-LN patients (69–79 % specificity, respectively, and 60 % sensitivity both). With this assay, a histological threshold (∼2x positive cut-off) can be further proposed to discriminate proliferative from membranous LN-A. Owing to the lack of standardization between manufacturers, the development of an international standard is expected and it is advised to adjust a positive/histological cut-off according to the local population.

Several criteria from this study support utility of coupling the urinary sCD163/creatinuria ratio and SLE-associated serological markers as LN biomarkers based on statistical analysis (e.g., logistic regression, correlation) and distinct associations with histological presentation, renal activity, and ESKD evolution. However and not tested in this study, the additional effect between serological and urinary biomarkers on LN progression needs to be further evaluated, and for that future and multicentric studies may help to answer this question.

In conclusion, and although serological markers and the urinary sCD163/creatinuria ratio independently provide an added value to monitor LN, additional non-invasive biomarkers have to be proposed for diagnosis and monitoring of LN in the near future. High-throughput technologies such as protein arrays, mass spectrometry-based proteomic analysis, and single-cell sequencing have already highlighted a long list of potential biomarkers, including the urinary sCD163/creatinuria ratio evaluated here, but their transfer to the clinical practice needs to be carefully validated as there is a need for accurate and robust biomarkers [40]. For that a consensus proposal from a group of experts is required based on well-established criteria (e.g. AUC>0.8–0.9, p < 0.05) and validation in cohorts as recently proposed [41].

## Funding

This research did not receive any specific grant from funding agencies in the public, commercial, or not-for-profit sectors.

## CRediT authorship contribution statement

**Yves Renaudineau:** Writing – review & editing, Writing – original draft, Validation, Project administration, Methodology, Formal analysis, Data curation, Conceptualization. **Dominique Chauveau:** Writing – review & editing, Data curation. **Stanislas Faguer:** Writing – review & editing, Data curation. **Antoine Huart:** Writing – review & editing, Data curation. **David Ribes:** Writing – review & editing, Data curation. **Gregory Pugnet:** Writing – review & editing, Data curation. **Laurent Sailler:** Writing – review & editing, Data curation. **Thibaut Jamme:** Writing – review & editing, Data curation. **Emmanuel Treiner:** Writing – review & editing, Data curation. **Françoise Fortenfant:** Writing – review & editing, Data curation. **Chloé Bost:** Writing – review & editing, Resources, Data curation. **Caroline Carlé:** Writing – review & editing, Data curation. **Julie Belliere:** Writing – review & editing, Writing – original draft, Project administration, Data curation, Conceptualization.

## Declaration of competing interest

The authors declare that they have no known competing financial interests or personal relationships that could have appeared to influence the work reported in this paper.

## Data Availability

Data will be made available on request.

## References

[1] Anders H.J. (2020). Lupus nephritis. Nat. Rev. Dis. Prim..

[2] Renaudineau Y. (2023). Immunological and translational key challenges in systemic lupus erythematosus: a symposium update. J Transl Autoimmun.

[3] Bajema I.M. (2018). Revision of the International Society of Nephrology/Renal Pathology Society classification for lupus nephritis: clarification of definitions, and modified National Institutes of Health activity and chronicity indices. Kidney Int..

[4] Fernandes das Neves M., Irlapati R.V., Isenberg D. (2015). Assessment of long-term remission in lupus nephritis patients: a retrospective analysis over 30 years. Rheumatology.

[5] Seret G. (2013). Homozygous FCGR3A-158F mutation is associated with delayed B-cell depletion following rituximab but with preserved efficacy in a patient with refractory lupus nephritis. Clin Kidney J.

[6] Gatto M. (2022). Clinical and histological findings at second but not at first kidney biopsy predict end-stage kidney disease in a large multicentric cohort of patients with active lupus nephritis. Lupus Sci Med.

[7] Xu J. (2020). Acute kidney disease increases the risk of post-kidney biopsy bleeding complications. Kidney Blood Press. Res..

[8] Palsson R. (2020). Bleeding complications after percutaneous Native kidney biopsy: results from the Boston kidney biopsy cohort. Kidney Int Rep.

[9] Renaudineau Y. (2006). Association of alpha-actinin-binding anti-double-stranded DNA antibodies with lupus nephritis. Arthritis Rheum..

[10] Croquefer S. (2005). The ananti-alpha-actinin test completes ananti-DNA determination in systemic lupus erythematosus. Ann. N. Y. Acad. Sci..

[11] Seret G. (2012). Mesangial cell-specific antibodies are central to the pathogenesis of lupus nephritis. Clin. Dev. Immunol..

[12] Tusseau M. (2022). DNASE1L3 deficiency, new phenotypes, and evidence for a transient type I IFN signaling. J. Clin. Immunol..

[13] Hartl J. (2021). Autoantibody-mediated impairment of DNASE1L3 activity in sporadic systemic lupus erythematosus. J. Exp. Med..

[14] Panda A.K., Ravindran B., Das B.K. (2016). CR1 exon variants are associated with lowered CR1 expression and increased susceptibility to SLE in a Plasmodium falciparum endemic population. Lupus Sci Med.

[15] Richoz N. (2022). Distinct pathogenic roles for resident and monocyte-derived macrophages in lupus nephritis. JCI Insight.

[16] Endo N. (2016). Urinary soluble CD163 level reflects glomerular inflammation in human lupus nephritis. Nephrol. Dial. Transplant..

[17] Zhang T. (2020). Association of urine sCD163 with proliferative lupus nephritis, Fibrinoid necrosis, cellular crescents and intrarenal M2 macrophages. Front. Immunol..

[18] Trouw L.A. (2004). Anti-C1q autoantibodies deposit in glomeruli but are only pathogenic in combination with glomerular C1q-containing immune complexes. J. Clin. Invest..

[19] Aringer M. (2019). 2019 European League against Rheumatism/American College of Rheumatology classification criteria for systemic lupus erythematosus. Ann. Rheum. Dis..

[20] Gladman D.D., Ibanez D., Urowitz M.B. (2002). Systemic lupus erythematosus disease activity index 2000. J. Rheumatol..

[21] Levey A.S. (2007). Expressing the Modification of Diet in Renal Disease Study equation for estimating glomerular filtration rate with standardized serum creatinine values. Clin. Chem..

[22] Carle C. (2022). Lupus band test can be used in combination with anti-chromatin antibodies and complement analysis to predict transition from cutaneous to systemic lupus. Clin. Immunol..

[23] Bories E. (2022). Myositis-specific autoantibodies in clinical practice: improving the performance of the immunodot. Semin. Arthritis Rheum..

[24] Bost C. (2021). Combining multi-antigenic immunodot with indirect immunofluorescence on HEp-2 cells improves the diagnosis of systemic sclerosis. Clin. Immunol..

[25] Puissant-Lubrano B. (2022). The oxygen carrier M101 alleviates complement activation, which may be beneficial for donor organ preservation. Front. Immunol..

[26] O'Reilly V.P. (2016). Urinary soluble CD163 in active renal vasculitis. J. Am. Soc. Nephrol..

[27] Moran S.M. (2021). The clinical application of urine soluble CD163 in ANCA-associated vasculitis. J. Am. Soc. Nephrol..

[28] Mejia-Vilet J.M. (2020). Urinary soluble CD163: a novel noninvasive biomarker of activity for lupus nephritis. J. Am. Soc. Nephrol..

[29] Levey A.S. (2020). Measured and estimated glomerular filtration rate: current status and future directions. Nat. Rev. Nephrol..

[30] Olmes G. (2016). CD163+ M2c-like macrophages predominate in renal biopsies from patients with lupus nephritis. Arthritis Res. Ther..

[31] Huang Y.J. (2022). Urine soluble CD163 is a promising biomarker for the diagnosis and evaluation of lupus nephritis. Front. Immunol..

[32] David C. (2020). Soluble CD163 is a biomarker for accelerated atherosclerosis in systemic lupus erythematosus patients at apparent low risk for cardiovascular disease. Scand. J. Rheumatol..

[33] Nishino A. (2019). Usefulness of soluble CD163 as a biomarker for macrophage activation syndrome associated with systemic lupus erythematosus. Lupus.

[34] Nakayama W. (2012). CD163 expression is increased in the involved skin and sera of patients with systemic lupus erythematosus. Eur. J. Dermatol..

[35] Yang G. (2021). Elevated soluble CD163 predicts renal function deterioration in lupus nephritis: a cohort study in Eastern China. J. Int. Med. Res..

[36] Botto M. (1998). Homozygous C1q deficiency causes glomerulonephritis associated with multiple apoptotic bodies. Nat. Genet..

[37] Walport M.J., Davies K.A., Botto M. (1998). C1q and systemic lupus erythematosus. Immunobiology.

[38] Eggleton P. (2014). Autoantibodies against C1q as a diagnostic measure of lupus nephritis: systematic review and meta-analysis. J. Clin. Cell. Immunol..

[39] Marzano A.V. (2022). Urticarial vasculitis: clinical and laboratory findings with a particular emphasis on differential diagnosis. J. Allergy Clin. Immunol..

[40] Rodriguez-Almaraz E. (2021). Something new about prognostic factors for lupus nephritis? A systematic review. Lupus.

[41] Tan G. (2021). Emerging molecular markers towards potential diagnostic panels for lupus. Front. Immunol..

